# Doxorubicin Release Controlled by Induced Phase Separation and Use of a Co-Solvent

**DOI:** 10.3390/ma11050681

**Published:** 2018-04-26

**Authors:** Seok Chan Park, Yue Yuan, Kyoungju Choi, Seong-O Choi, Jooyoun Kim

**Affiliations:** 1 Department of Anatomy and Physiology, Kansas State University, Manhattan, KS 66506, USA; schpark@ksu.edu (S.C.P.); kjchoi@vet.k-state.edu (K.C.); sochoi@ksu.edu (S.-O.C.); 2 Department of Textiles Engineering, Chemistry and Science, North Carolina State University, Raleigh, NC 27695, USA; yyuan14@ncsu.edu; 3 Nanotechnology Innovation Center of Kansas State, Kansas State University, Manhattan, KS 66506, USA; 4 Department of Textiles, Merchandising and Fashion Design, Seoul National University, Seoul 08826, Korea; 5 Research Institute of Human Ecology, Seoul National University; Seoul 08826, Korea

**Keywords:** poly(lactic acid), polyvinylpyrrolidone, dimethyl sulfoxide, doxorubicin, phase separation, co-solvent, porogen, drug release, electrospinning

## Abstract

Electrospun-based drug delivery is emerging as a versatile means of localized therapy; however, controlling the release rates of active agents still remains as a key question. We propose a facile strategy to control the drug release behavior from electrospun fibers by a simple modification of polymer matrices. Polylactic acid (PLA) was used as a major component of the drug-carrier, and doxorubicin hydrochloride (Dox) was used as a model drug. The influences of a polar co-solvent, dimethyl sulfoxide (DMSO), and a hydrophilic polymer additive, polyvinylpyrrolidone (PVP), on the drug miscibility, loading efficiency and release behavior were investigated. The use of DMSO enabled the homogeneous internalization of the drug as well as higher drug loading efficiency within the electrospun fibers. The PVP additive induced phase separation in the PLA matrix and acted as a porogen. Preferable partitioning of Dox into the PVP domain resulted in increased drug loading efficiency in the PLA/PVP fiber. Fast dissolution of PVP domains created pores in the fibers, facilitating the release of internalized Dox. The novelty of this study lies in the detailed experimental investigation of the effect of additives in pre-spinning formulations, such as co-solvents and polymeric porogens, on the drug release behavior of nanofibers.

## 1. Introduction

Over recent decades, electrospinning has been used as a versatile means of fabricating submicron fibers. A typical laboratory set-up of the electrospinning process consists of high voltage power supply, syringe, needle and conducting collector [1,2,3]. This old technique invented in the 1930s has regained academic and industrial attention with a growing interest in nanotechnology. As the fibrous web of nanofibers resembles the native extracellular matrix in its physical structure, electrospun nanofibers have been widely employed particularly in biomedical engineering. Especially, the high porosity of electrospun materials promotes the permeation of gases and nutrients [4,5,6] in tissue scaffolds, and high surface-to-volume ratio facilitates the adhesion and proliferation of cells [7,8]. With such characteristics, electrospun materials have become an attractive option for tissue engineering [9,10] and prospective drug delivery systems [8,11].

The main objective of drug delivery is to design a desired release profile while minimizing adverse side effects. To enhance the biocompatibility and reduce the major immune responses, a wide range of biocompatible polymers have been tested as carrier polymers [12,13,14,15,16,17,18]. For the fiber-based drug delivery, a drug is usually mixed with the carrier polymer in a pre-spinning solution. After the fibers are spun, drug molecules may exist as dispersions in a polymer matrix or be internalized like a monolithic polymer solution depending on the compatibility between the drug molecules and the carrier polymer. The release kinetics of a drug delivery system can be controlled by altering the solubility and swelling of carrier polymers. In the erosion-controlled system, the release can be promoted by the increased degradability and solubility of the carrier. In the diffusion-controlled system, the release can be promoted by the wettability and swelling of the carrier [19]. As hydrophobic materials such as poly(lactic-co-glycolic acid) (PLGA) [20,21,22] and polylactic acid (PLA) [18,23,24] poorly swell and slowly degrade in the human body, these materials tend to show a slow release of drugs without bioaccumulation [25,26,27,28,29,30,31,32]. On the contrary, fast biodegrading polymers such as gelatin [14,15,16] and polyvinylpyrrolidone [33] tend to exhibit immediate release of incorporated drugs.

To adjust the degradability and wettability of a polymeric matrix, polymer blends [34,35,36] or block copolymers [21,37] can be formulated by selective material combinations. For example, adding a water-soluble/swellable polymer additive in a hydrophobic carrier system can significantly increase the drug release rate [38]. In addition to modifying the system formulations, distinct release kinetics can be achieved by utilizing multilayered [35,39,40,41] or porous structures [41,42,43]. Environmental stimulations such as pH, temperature, light, electrical field, and magnetic field [44,45,46,47,48,49,50,51,52,53] can also be used to manipulate the release at the specific target sites. While many of those methods often require sophisticated process modifications, in practical applications it is desirable to establish a simple strategy to manipulate the drug release.

The objective of this study is to develop a facile means of controlling drug release behavior by modifying the formulation of pre-spinning solutions. Polylactic acid (PLA) was used as a main polymeric drug-carrier, and doxorubicin HCl (Dox), a hydrophilic form of Dox, was used as a model drug. By adding 20 wt % of polyvinylpyrrolidone (PVP) in PLA matrix, the phase separation was purposely induced. Due to the hydrophilic nature of PVP, PVP acted as a porogen in the hydrophobic PLA matrix. The selective dissolution of PVP domains during the release stage created pores in the matrix, influencing the diffusivity of Dox in the medium. Furthermore, dimethyl sulfoxide (DMSO) was employed as a co-solvent of Dox, and its role on drug-polymer compatibility and release characteristics was investigated. The effect of DMSO on Dox-miscibility in a hydrophobic polymer was discussed using the concept of solubility parameters. The novelty of this study is in the detailed experimental investigation of drug release behavior in the presence of a hydrophilic co-solvent and a polymeric porogen. The findings of this research would provide a simple guide of controlling drug release behavior in a fiber-based drug delivery system, with a minimal modification of pre-spinning formulations.

## 2. Materials and Methods 

### 2.1. Materials 

Polylactic acid (PLA) resin (Ingeo 4043D, NatureWorks), 98% L-lactide, with weight average molecular weight of 111 kg/mol was purchased from NatureWorks (Minnetonka, MN, USA). Polyvinylpyrrolidone (PVP) with average molecular weight of 1300 kg/mol was purchased from Alfa Aesar (Haverhill, MA, USA). Dimethyl sulfoxide (DMSO) and dimethylformamide (DMF) were purchased from Sigma-Aldrich (St. Louis, MO, USA). Doxorubicin hydrochloride (99%, Dox-HCl) was purchased from MedKoo Biosciences (Morrisville, NC, USA). Solvents, phosphate buffered saline (PBS, pH 7.4) were purchased from Thermo Fisher (Waltham, MA, USA), and other chemicals were purchased from Fisher Scientific (Hampton, NH, USA). 

### 2.2. Preparation of Drug-Loaded Fiber Webs 

For PLA electrospinning, 9% (*w/v*), the PLA solution was prepared in a 1:1 ratio of dichloromethane (DCM) and dimethyl formamide (DMF). For PVP blending, 16% (*w/v*) of PVP solution was prepared in DMF, and then this solution was mixed with the PLA solution in 80:20 weight ratio. For electrospinning (Spraybase®, Dublin, Ireland), a grounded aluminum collector was placed in front of a 22-gauge needle at the distance of 10 cm, and fibers were spun horizontally toward the collector rotating at 100 rpm. The feeding rate of polymer solution was 1.5 mL/h. The applied voltage was adjusted to 11 kV~14 kV to produce non-beaded fibers. 

For drug loading, doxorubicin hydrochloride (Dox) was added in the polymer solutions (PLA and PLA/PVP blend) before electrospinning by two methods. One of the methods was to mix the drug particles directly to a PLA or a PLA/PVP pre-spinning solution, stirring at 100 rpm for 1 h at room temperature. The formulation for PLA with Dox pre-spinning solution was: Dox 0.02 g, PLA 0.4 g, 2 mL DCM, 2 mL DMF. The formulation for PLA/PVP mixture was: Dox 0.02 g, PLA 0.315 g (10% PLA solution 3.5 mL), 0.08 g PVP (16% PVP solution 0.5 mL). In the other method, Dox was firstly dissolved in DMSO by 2% (*w/v*), and 1 mL of Dox/DMSO solution was mixed with 4 mL of 9% (*w/v*) PLA solution (PLA-Dox(DMSO)) or 4 mL of PLA/PVP solution (PLA/PVP-Dox(DMSO)), respectively. The sample codes and descriptions are shown in Table 1.

### 2.3. Characterization 

Optical and fluorescence images of Dox-loaded webs were observed with an inverted fluorescent microscope system (Olympus IX73, Olympus Corporation, Tokyo, Japan). Images were analyzed by ImageJ software (version 1.46r, NIH, Bethesda, MD, USA). Fiber morphology was observed by a scanning electron microscope (Merlin Compact FE-SEM, Zeiss, Oberkochen, Germany) with Pt sputter coating for 200 s (MSC-101, JEOL, Tokyo, Japan). 

Static contact angles (CA) of 4 µL of distilled water on fibrous surfaces were measured by an optical tensiometer (Attension Theta, Biolin Scientific, Paramus, NJ, USA) at room temperature. 

### 2.4. Solubility Parameter 

To explain the compatibility of polymers and solvents, Hansen solubility parameters from reference values were used (Table 2). The Hansen solubility parameter in Equation (1) is comprised of three parameters: energy from dispersion bonds between molecules, δ_d_; dipolar intermolecular force between molecules, δ_p_; and the hydrogen bonds between molecules, δ_h_. The solubility parameter of mixture solvent was calculated using Equation (2). To estimate the compatibility between two components, the interaction parameter, R, was calculated by Equation (3). The calculated values of R’s are presented in Table 3, where a smaller R represents a higher compatibility between components.

(1) $$\delta_{t}=\sqrt{{\left(\delta_{d1}-\delta_{d2}\right)}^{2}+{\left(\delta_{p1}-\delta_{p2}\right)}^{2}+{\left(\delta_{h1}-\delta_{h2}\right)}^{2}},$$

(2) $$\delta_{\mathrm{mix}}=f_{1}\cdot \delta_{1}+f_{2}\cdot \delta_{2},$$

(3) $$R=\sqrt{4{\left(\delta_{d1}-\delta_{d2}\right)}^{2}+{\left(\delta_{p1}-\delta_{p2}\right)}^{2}+{\left(\delta_{h1}-\delta_{h2}\right)}^{2}},$$

δ_t_ (MPa^1/2^): total solubility parameter; δ_d_ (MPa^1/2^): dispersion force component; δ_p_ (MPa^1/2^): polar force component; δ_h_ (MPa^1/2^): hydrogen-bonding component; δ_1_ and δ_2:_ solubility of the components 1 and 2, respectively; δ_mix_ (MPa^1/2^): solubility parameter of the mixture of components 1 and 2; $f_{1}$ and $f_{2}$: volume ratio of the components 1 and 2; R (MPa^1/2^): interaction parameter.

### 2.5. Phase Separation

Phase separation of PLA and PVP blends was observed to investigate the drug distribution between PLA and PVP regions. The 5 µL pre-spinning solutions with and without drug were loaded between two glass coverslips, and observed under the inverted fluorescent microscope system. The solution compositions for the phase separation study were maintained the same as the pre-spinning solutions to simulate the phase separation occurring during web formation. 

### 2.6. Loading Content (LC) and Loading Efficiency (LE)

Drug loading content (LC%) was determined by the weight percentage of a drug in a fiber web (Equation (4)). For this measurement, drug-loaded fiber webs were cut into rectangular strips and weighed. The samples were completely dissolved in 2.5 ml of chloroform and mixed vigorously with 5 ml of deionized water (DI water). The mixtures were stored in dark overnight for spontaneous phase separation of organic and aqueous phases. The supernatant was taken after centrifugation at 4696 × g and diluted 10-fold for Ultraviolet/Visible (UV/Vis) spectroscopy (Synergy H1 Hybrid Multi-Mode Reader, BioTek, Winooski, VT, USA). The number of drugs in the web were calculated from the triplicate measurements of absorbance of Dox at 480 nm. Loading efficiency (LE%) of a drug-loaded fiber web was calculated by Equation (5).

(4) $$\mathrm{LC}\;\left(\%\right)=\frac{\mathrm{weight}\;\mathrm{of}\;\mathrm{drug}\;\mathrm{loaded}\;\mathrm{in}\;\mathrm{the}\;\mathrm{web}\;\left(\mathrm{mg}\right)}{\mathrm{weight}\;\mathrm{of}\;\mathrm{the}\;\mathrm{drug}\;\mathrm{loaded}\;\mathrm{fiber}\;\mathrm{web}\;\left(\mathrm{mg}\right)}\times 100\;\left(\%\right),$$

(5) $$\mathrm{LE}\;\left(\%\right)=\frac{\mathrm{weight}\;\mathrm{of}\;\mathrm{drug}\;\mathrm{loaded}\;\mathrm{in}\;\mathrm{the}\;\mathrm{web}\;\left(\mathrm{mg}\right)\;}{\mathrm{weight}\;\mathrm{of}\;\mathrm{drug}\;\mathrm{initally}\;\mathrm{added}\;\mathrm{in}\;\mathrm{the}\;\mathrm{polymer}\;\mathrm{solution}\;\left(\mathrm{mg}\right)}\times 100\;\left(\%\right).$$

### 2.7. In-Vitro Drug Release Analysis

About 20 mg of a drug-loaded web was immersed in 3.5 mL of phosphate buffered saline (PBS, pH 7.4) at 37 °C and was stirred at 100 rpm using a vertical diffusion cell (Perme Gear, Hellertown, PA, USA). At each sampling point, the full amount of 3.5 mL of drug-released PBS medium was replaced with fresh 3.5 mL PBS. To measure the amount of drug released from the fiber composites, the fluorescence intensity of doxorubicin (590 nm) was measured using a UV-visible microplate reader (Synergy H1 Hybrid Multi-Mode Reader, BioTek) with an excited wavelength of 480 nm. The amount of drug released from the web at each sampling interval was quantified by the calibration curve for fluorescence intensities of Dox standard concentrations. The cumulative percentage of released drug at each sampling point was calculated by Equation (6). All experiments were conducted with at least three replications.

(6) $$\mathrm{cumulative}\;\mathrm{drug}\;\mathrm{release}\;\left(\%\right)=\frac{\sum_{t_{0}}^{t_{n}}\mathrm{weight}\;\mathrm{of}\;\mathrm{drug}\;\mathrm{released}\;\left(\mathrm{mg}\right)}{\mathrm{weight}\;\mathrm{of}\;\mathrm{drug}\;\mathrm{in}\;\mathrm{the}\;\mathrm{web}\;\left(\mathrm{mg}\right)\;}\times 100\;\left(\%\right),$$

t_0_: The initial collection point at release time = 0 h; t_n_: The *n*^th^ collection point at release time = *n* h.

## 3. Results

### 3.1. Drug Localization in the Drug-Carrier

**Figure 1:**
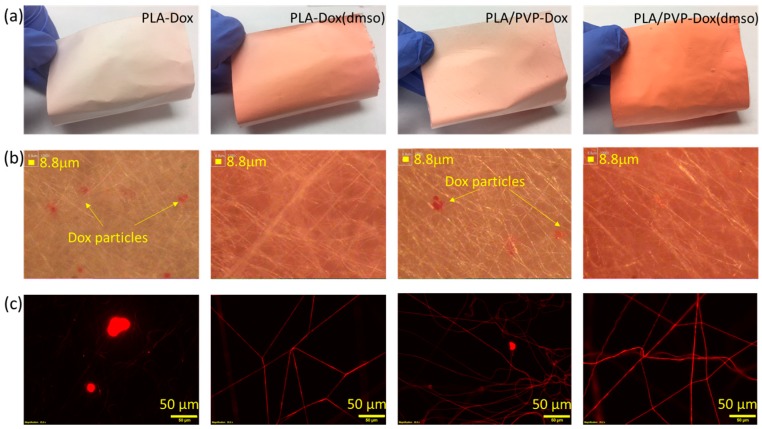
Doxorubicin-loaded fiber webs. (**a**) Photographic images of drug-loaded webs; (**b**) optical images displaying drug aggregates; (**c**) fluorescence images with Dox-loaded webs.

The influence of immiscible polymer blending on drug localization in the fiber-based drug-carrier was observed by optical and fluorescence microscopies (Figure 1). Doxorubicin HCl used in this study has hydrophilic nature and can be dissolved in hydrophilic solvents such as water and DMSO. When Dox was directly added to a hydrophobic PLA pre-spinning solution (PLA in DCM/DMF), Dox was not soluble; instead, its particles were aggregated and precipitated in the solution. Due to the lack of compatibility between Dox and PLA, Dox-incorporating fibers produced large Dox aggregates as observed from the optical and fluorescence images in Figure 1b,c. Comparing fluorescence images of PLA-Dox and PLA/PVP-Dox (Figure 1c), PLA-Dox fibers were rarely fluorescent, while PLA/PVP-Dox fibers were fluorescent resulting from the incorporated Dox. For PLA/PVP-Dox samples, Dox appears to be mostly internalized in the hydrophilic PVP domains rather than PLA domains. From the fact that Dox particles still remained in the PLA/PVP-Dox web, 20% of PVP content may not be sufficient to fully internalize the added Dox (loading content ~3.1%).

When Dox was dissolved in DMSO, Dox was homogeneously internalized in the fibers, leaving little Dox particles, and the fibers produced more saturated color. From the microscopic images, when Dox was dissolved in DMSO, the miscibility of Dox in hydrophobic PLA appeared to be significantly improved, with the enhanced interaction between Dox/DMSO and PLA solution. Likewise, PLA/PVP-Dox(DMSO) exhibited good compatibility between Dox and PLA with little Dox particles. From the solubility parameters of solvents, the interaction parameter R between PVP and DMSO was calculated to be about 4.1 (Table 3), which is smaller than that of PLA and DMSO; this implies that DMSO is more compatible with PVP than with PLA. Thus, in a PLA/PVP blend, DMSO would favor partitioning in PVP over PLA. The compatibility among Dox, PLA and PVP was further examined by casting polymeric films.

### 3.2. Phase Separation Between PLA and PVP

**Figure 2:**
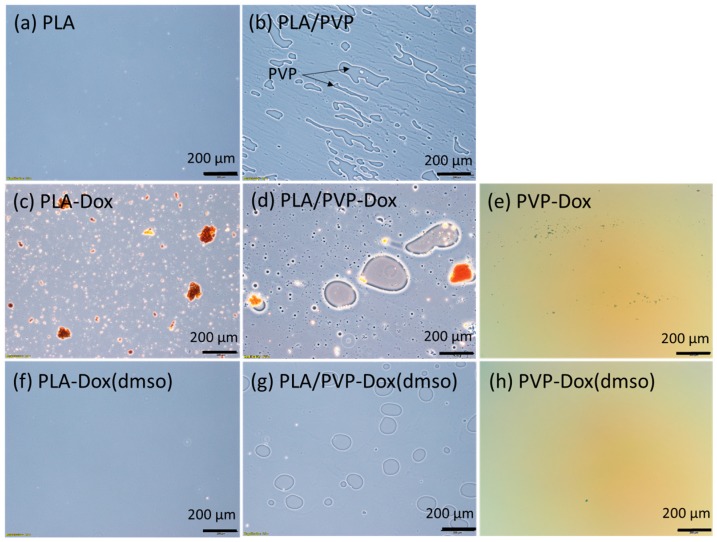
Microscopy images of polylactic acid (PLA) solution with homogenous single phase and PLA/polyvinylpyrrolidone (PVP) solution with phase separation.

**Figure 3:**
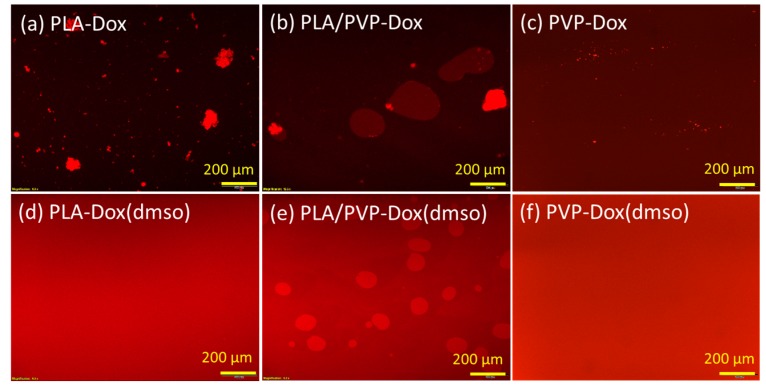
Fluorescence microscopy images of PLA-doxorubicin hydrochloride (Dox), PLA-Dox(dimethyl sulfoxide (DMSO)), PLA/PVP-Dox and PLA/PVP-Dox(DMSO) pre-spinning solutions casted on glass slides.

The phase separation was observed from the polymer blends loaded on glass coverslips (Figure 2) [34,35]. For PLA/PVP blends, 20% of PVP created its domains in the blend matrices by phase separation, due to the lack of compatibility between PLA and PVP (Figure 2b). When Dox was added to a PLA/PVP blend without DMSO, Dox was either dissolved in PVP domains or formed aggregates in the bulk (Figure 2d and Figure 3b, PLA/PVP-Dox). When Dox was dissolved in DMSO, Dox was well-blended in both PVP and PLA domains, while a clear phase separation was still observed (Figure 2g and Figure 3e). When DMSO was added, the size of PVP domains was considerably reduced, producing smaller emulsions due to the enhanced stability of the mixture.

For a PLA-only matrix, Dox particles formed large aggregates (Figure 2c and Figure 3a, PLA-Dox); with the aid of DMSO, Dox was homogeneously dissolved like a monolithic matrix (Figure 2f and Figure 3d, PLA-Dox(DMSO)). Little or no fluorescence was observed from PLA background (Figure 1c), implying Dox was not well internalized in PLA matrix without an aid of DMSO. The hydrophilic Dox itself (without DMSO) was blended better into PVP (Figure 2e and Figure 3c) than into PLA, yet small Dox particles remained in the PVP matrix. DMSO enhanced the miscibility of Dox in the PVP-only matrix also, leaving little particles in the bulk matrix (Figure 2h and Figure 3f).

### 3.3. Morphological Influence of Fibers Before and After Immersion in Water

**Figure 4:**
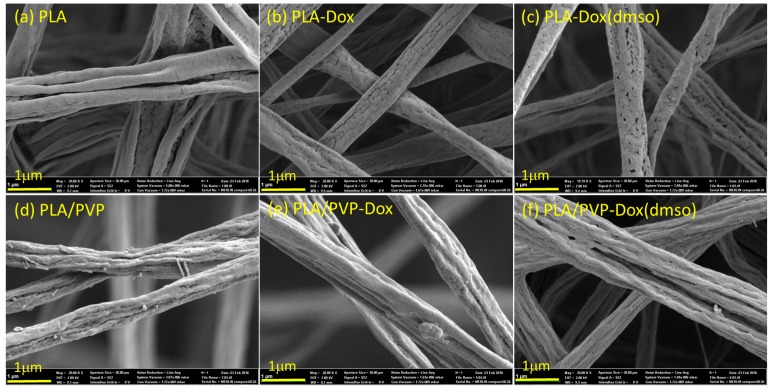
SEM images of doxorubicin-loaded fibers.

The morphology of fiber webs is shown in Figure 4. When DMSO was added to PLA and PLA/PVP, small pores were formed on the fiber surfaces; the size of pores ranged from tens of nanometers to about 300 nm when measured by ImageJ. As the volatility of DMSO (vapor pressure ~59 Pa at 20 °C) is considerably lower than that of DCM (vapor pressure ~47,400 Pa at 20 °C) and DMF (vapor pressure ~380 Pa at 20 °C), DMSO remains on a fiber surface longer than other solvents when fibers solidify upon solvent evaporation. When DMSO finally evaporates from the fiber surface, pores can be created on the surface, as observed in Figure 4c. 

**Figure 5:**
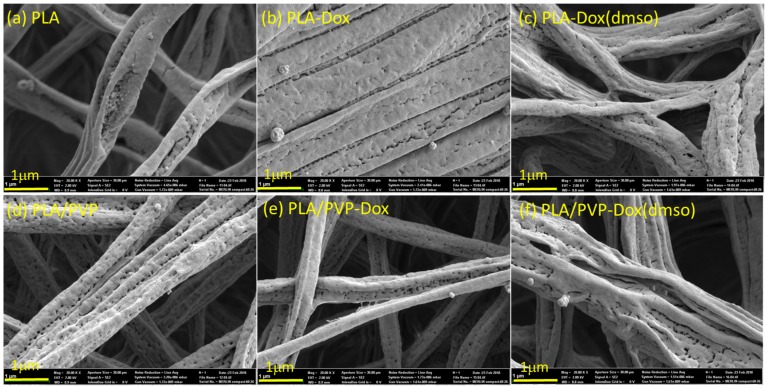
SEM images of doxorubicin-loaded fibers after immersion in water for 24 h.

To investigate the morphological changes of fibers with water immersion, fibrous webs were immersed in distilled water for 24 h at 37 °C and observed by SEM (Figure 5). For PLA/PVP fibers, the hydrophilic PVP acted as a porogen in hydrophobic PLA matrix, and after 24 h immersion, pores were created (in tens of nanometers to about 300 nm) on the fiber surface; on the other hand, PLA fibers did not show noticeable morphological changes. The influence of these pore formation on the Dox-release profile is discussed in a later section.

### 3.4. Wettability of Drug-Loaded Webs

**Figure 6:**
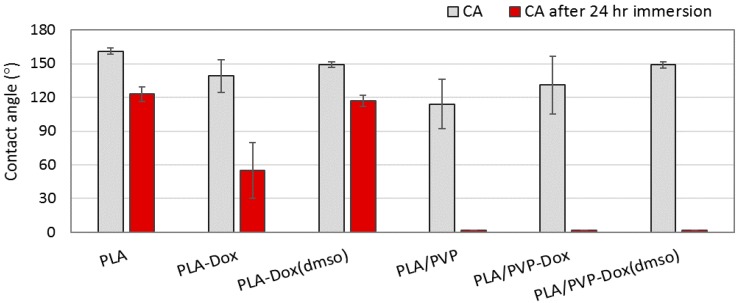
Contact angle of Dox-loaded fiber webs before and after immersion in water for 24 h.

The influence of PVP blending to PLA on wettability was examined by water contact angle (CA) measurement (Figure 6). The CA of pristine PLA web without drug loading was 160°. When Dox was added to PLA (PLA-Dox), CA decreased to about 139°. The wetting property was not uniform because the CA measurement varied depending on the presence of Dox aggregates on the measured locations. When Dox was uniformly blended in PLA matrix with the aid of co-solvent DMSO, surface wettability turned more uniform with CA about 148°. With the addition of PVP (PLA/PVP and PLA/PVP-Dox), CA dropped to 114°~131°; also, the CA meausrements had a large variation depending on the size of PVP domains on the surface. When DMSO was added to the PLA/PVP blend (PLA/PVP-Dox(DMSO)), surface homogeneity was improved and the measured CA was consistent (~149°).

To examine how the wettability changes with drug release, CA was measured after 24 h of water immersion at 37 °C. The immersed webs were completely dried before CA re-measurement. After the immersion, PLA without Dox loading (CA ~123°) and monolithic-like PLA-Dox(DMSO) (CA ~117°) maintained its hydrophobicity. However, wettability of PLA-Dox was considerably increased after the immersion. For all PVP blended samples, wettability was significantly enhanced after the immersion, where the water drop was immediately spread on the surface (CA ~0°). From Figure 5d–f, PVP domains of the fibers began to dissolve upon water exposure, creating pores. The dissolved PVP may have coated the fiber surface, turning the surface more hydrophilic. Also, the irregular surface roughness resulting from pores and wrinkles, combined with the increased hydrophilicity, contributed to further increase of wettability according to the Wenzel theory [57]. 

### 3.5. Loading Contents (LC) and Loading Efficiency (LE)

**Figure 7:**
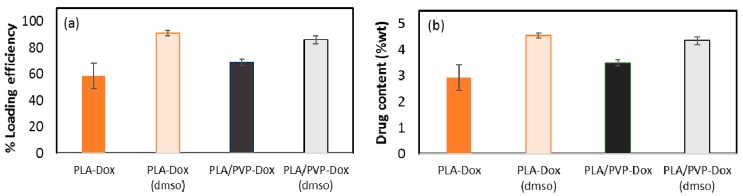
Loading content (**a**) and loading efficiency (**b**) of doxorubicin in the web.

The loading content (LC) and loading efficiency (LE) of Dox were examined (Figure 7). PLA-Dox, in which Dox was not homogeneously blended with PLA, showed only 59% LE, producing the actual drug content of 2.9% (*w/w*). When hydrophilic Dox was blended with PLA solution, the system was not stable; Dox formed aggregates and precipitated in the solution. The precipitates of Dox particles were visible at the bottom of the pre-spinning solution, and those Dox precipitates were not fully incorporated in the fibers during the spinning process. Thus, the incompatibility between the hydrophilic drug and the hydrophobic carrier polymer led to low drug loading efficiency and content. 

With DMSO, Dox was well mixed with the PLA matrix, and there were no precipitates formed. For PLA-Dox(DMSO), LE of Dox increased up to about 91%, forming a homogeneous, monolithic-like matrix with LC about 4.6%. When a hydrophilic PVP polymer additive was mixed with PLA matrix, Dox appeared to dissolve mostly in PVP domains (Figure 3b), demonstrating the favorable partitioning of a hydrophilic drug into a hydrophilic polymer. Compared to PLA-Dox, PLA/PVP-Dox showed the enhanced Dox loading with LE of about 69% (LC ~3.5%). When DMSO was used as a co-solvent of Dox in the PLA/PVP, the LE was about 86%. As the solubility parameter of DMSO is closer to PVP than PLA, it was expected that loading of Dox/DMSO would be more efficient in PLA/PVP than in PLA. However, the results did not show this trend; on the contrary, LE% of Dox/DMSO in PLA/PVP blend was slightly lower than PLA-Dox(DMSO). While the information is missing to explain this phenomenon, it can be concluded that the overall loading efficiency was enhanced by the addition of hydrophilic polymer additive and the hydrophilic co-solvent. 

### 3.6. In-Vitro Dox Release

**Figure 8:**
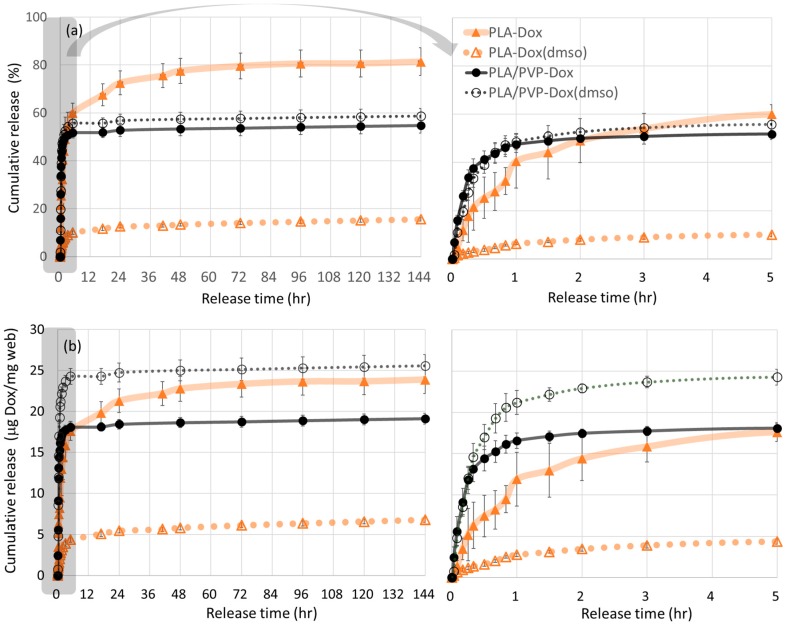
In-vitro 6-day release profile of doxorubicin from PLA and PLA/PVP webs. (**a**) Cumulative % release of Dox (left, release during 144 h; right, enlarged for 5 h); (**b**) cumulative released amount (mg) of Dox (left, release during 144 h; right, enlarged for 5 h).

From the in-vitro drug release in Figure 8, Dox dissolved in PLA with DMSO exhibited the distinct sustained release. The use of DMSO as a co-solvent considerably improved the miscibility of Dox and PLA, producing a homogeneous monolithic-like matrix. As PLA is rarely swelling in water, the internalized Dox in the monolithic-like fiber can hardly be diffused out from the polymer matrix; thus, the release of internalized Dox would be dependent mostly on PLA degradation. As is shown in SEM images of Figure 5, PLA fiber morphology had little changes after 24 h water immersion. As PLA degradation takes months of period, the release of Dox from the monolithic-like fibers would take as long as several months. 

Rapid release with initial burst profile was shown by PLA-Dox, PLA/PVP-Dox, and PLA/PVP-Dox(DMSO) fibers. PLA-Dox, in which Dox formed aggregates, exhibited faster release in the first 5 h (about 60%). The release during this period is attributed to the direct dissolution of Dox particles that are exposed to web surface. The Dox particles mostly existed as aggregates on the fiber surfaces due to the lack of compatibility between Dox and PLA. The release profile of PLA-Dox exhibited a large variation as a result of inhomogeneous dispersions of Dox crystals over the fiber surfaces. Dox in PLA was released up to about 80% of the total content till 3 days, which indicates that about 80% of Dox is distributed near the surface of PLA fibers. The remaining 20% Dox may exist inside PLA matrix, and the release of this portion would occur with PLA degradation. 

PLA/PVP-Dox and PLA/PVP-Dox(DMSO) displayed similar release profiles. As Dox was preferentially dissolved in PVP over PLA domains, the release in the early stage is mostly attributed to PVP dissolution in PBS solution. The preferential distribution of Dox/DMSO in PVP over PLA domains can be validated by the lower interaction parameter, R between DMSO and PVP (4.1 MPa^1/2^) than R between DMSO and PLA (7.7 MPa^1/2^) (Table 3). The higher release rate of PLA/PVP than that of PLA suggests that the selective dissolution of water-soluble PVP is a predominant factor of the release. Particularly, the burst release within 1 h is attributed to the fast dissolution of PVP domains with the incorporated Dox (Figure 8, enlarged plots); this selective dissolution is well observed from the pores created on the fiber surface after 24 h immersion (SEM images in Figure 5). For PLA/PVP-Dox(DMSO), 55% of cumulative release during the initial 3 h is attributed to the dissolution of PVP domains containing Dox. About 40–45% of the total drug content distributed in PLA domains would be released along with PLA degradation. 

In practical drug delivery applications, the amount of drug released, rather than the percentage of the loaded, may be more important. Considering the loading efficiency and the release rates (%), the Dox amount (µg) released from 1 mg of web was calculated (Figure 8b). From the results, PLA/PVP-Dox(DMSO) showed the highest amount of drug release (µg Dox/mg web) during the first 24 h of release. While the released percentage of the total loaded was similar between PLA/PVP-Dox and PLA/PVP-Dox(DMSO), the total released amount (µg Dox/mg web) was considerably higher for PLA/PVP-Dox(DMSO) (~0.5 mg) than PLA/PVP-Dox (~0.38 mg), due to the enhanced loading efficiency with DMSO. With low LE% of PLA-Dox (2.9%), the amount of Dox released from PLA-Dox was lower than that of PLA/PVP-Dox(DMSO). The amount of Dox released from PLA-Dox(DMSO) was still the lowest while the LE% was as high as 91%. Regarding in-vivo perspectives, PLA/PVP-Dox(DMSO) may also be advantageous for reaching the necessary therapeutic levels faster than others tested by releasing the highest dosage in the first 5 h. As Dox incorporated in PLA would be released with PLA degradation, if enzymatic degradation of PLA is triggered in the body, Dox would be released faster under in-vivo conditions compared to in-vitro release. For effective treatments, it is necessary to load a sufficient amount of drugs and release at a controlled rate. Our data suggest that the addition of DMSO in the polymeric system increased the Dox loading by enhancing miscibility/compatibility in the polymeric matrix and could alter drug release rates.

While the compatibility of Dox both in PVP and PLA was enhanced with the use of DMSO, the effect on the release rate was completely different. For PLA-Dox(DMSO), the addition of DMSO lowered the release rates despite the improved Dox miscibility in PLA matrix, indicating that increased drug miscibility in a hydrophobic, slowly-degrading polymer matrix would retard the release of drugs. On the contrary, the use of DMSO in PLA/PVP blend further promoted the loading efficiency and release. The increased drug miscibility and solubility in a hydrophilic, fast-degrading hydrophilic polymer accelerated the drug release. The results demonstrate that the release of doxorubicin can be conveniently manipulated by the selection of polymer additives and solvents system. 

## 4. Conclusions

The release of a hydrophilic drug, doxorubicin hydrochloride (Dox), incorporated in electrospun PLA fibers was investigated along with drug loading efficiency to develop a facile strategy for controlling drug release from fiber-based drug delivery systems. Influences of miscibility and compatibility between the drug and polymer matrix on the loading and release properties were studied by adding a co-solvent and/or a hydrophilic polymer additive. A polar organic solvent, DMSO, and water-soluble polymer, PVP, were employed to enhance the miscibility of drug in a hydrophobic carrier polymer. In general, drug loading efficiency (LE) was improved by the addition of DMSO and PVP. The use of DMSO enabled the homogeneous incorporation of Dox, as well as higher drug loading, within the electrospun fibers. 

Distinct drug release behaviors were demonstrated by use of DMSO and PVP. In the monolithic PLA fibers, the addition of DMSO helped homogeneous encapsulation of Dox in PLA matrix, and this led to the delayed Dox release. Without DMSO, Dox formed inhomogeneous dispersions on the PLA fiber surfaces, resulting in the burst release. When PVP was added in the polymer system, preferable partitioning of Dox into the PVP domains occurred, and this resulted in the enhanced loading of Dox in PLA/PVP fibers. The hydrophilic PVP additive acted as a porogen in the PLA/PVP system, and the porous morphology formed by the selective dissolution of PVP promoted the diffusion of the drug in a short time period. 

In this study, doxorubicin release from nanofibers was manipulated by the addition of a hydrophilic porogen, the induced phase separation, and the use of co-solvent. The results suggest the importance of individual roles of solvent systems and polymer additives on drug release profiles. The addition of a compatible solvent to a polymer matrix enhanced drug loading efficiency and incorporation. However, the effect of drug-carrier miscibility on release rate was different for polymer types. The enhanced drug miscibility in a hydrophobic PLA matrix delayed the release, while the enhanced drug miscibility in a hydrophilic PVP matrix accelerated the release. The experimental investigation of this study confirmed the significance of the pre-spinning formulation in controlling the drug release rates. An extended study is recommended to test biocompatibility, in-vitro and in-vivo cancer cell viability to validate the effectiveness of the developed system.

## Figures and Tables

**Table 1:** Sample descriptions.

Code	Description
PLA	PLA web
PLA/PVP	PLA/PVP blend web (80:20 weight ratio)
PLA-Dox	Dox-loaded PLA web, Dox was mixed with PLA pre-spinning solution
PLA/PVP-Dox	Dox-loaded PLA/PVP web, Dox was mixed with PLA/PVP pre-spinning solution
PLA-Dox(DMSO)	Dox-loaded PLA web, Dox dissolved in DMSO was mixed with PLA pre-spinning solution
PLA/PVP-Dox(DMSO)	Dox-loaded PLA/PVP web, Dox dissolved in DMSO was mixed with PLA/PVP pre-spinning solution

**Table 2:** Hansen solubility parameters.

Component	δ_t_ (MPa^1/2^)	δ_d_ (MPa^1/2^)	δ_p_ (MPa^1/2^)	δ_h_ (MPa^1/2^)
PLA [54]	21.2	17.5	9.5	7.3
PVP [55]	24.3	18.8	13.4	7.5
DMSO [56]	26.7	18.4	16.4	10.2
DCM [56]	20.2	18.2	6.3	6.1
DMF [56]	24.9	17.4	13.7	11.3
DCM/DMF *	22.2	17.8	10.0	8.7

* Calculated value for 1:1 ratio of DCM:DMF.

**Table 3:** Interaction parameters R between polymer and solvents.

R (MPa^1/2^)	DMSO	DCM	DMF	DCM/DMF
PLA	7.7	3.7	5.8	1.6
PVP	4.1	7.3	4.7	4.1
DMSO	0	10.9	3.5	6.7
